# Protective Effect of *Meretrix meretrix* Oligopeptides on High-Fat-Diet-Induced Non-Alcoholic Fatty Liver Disease in Mice

**DOI:** 10.3390/md16020039

**Published:** 2018-01-23

**Authors:** Fangfang Huang, Jiajia Wang, Fangmiao Yu, Yunping Tang, Guofang Ding, Zuisu Yang, Yu Sun

**Affiliations:** 1 School of Food Science and Pharmacy, Zhejiang Provincial Key Engineering Technology Research Center of Marine Biomedical Products, Zhejiang Ocean University, Zhoushan 316000, China; gracegang@126.com (F.H.); 1354193287@139.com (J.W.); ymyu@zjou.edu.cn (F.Y.); tangyunping1985@163.com (Y.T.); dinggf2007@163.com (G.D.); 2 Zhejiang Provincial Key Engineering Technology Research Center of Marine Biomedical Products, Zhejiang Ocean University Donghai Science and Technology College, Zhoushan 316000, China

**Keywords:** non-alcoholic fatty liver disease, *Meretrix meretrix* oligopeptides, NF-κB anti-inflammation signaling pathways

## Abstract

The present study investigated the effects of MMO (*Meretrix meretrix* oligopeptides) on mice fed a high-fat diet. Mice were fed either a normal control diet (NC) or a high-fat diet (HFD) without or with MMO (50 mg/kg or 250 mg/kg) for four weeks. Levels of ALT, AST, liver tissue GSH-Px, and SOD activities, MDA levels were measured using commercially available kits; HE staining was performed to analyze pathologic changes of the liver; a TEM assay was performed to measure the ultrastructural alterations of the mitochondria, and Western blotting was performed to detect the expression of gene proteins related to lipid metabolism, inflammation, and liver apoptosis. After six weeks, body weight, ALT, AST, and MDA levels were significantly increased, and GSH-Px levels and SOD activities were significantly decreased in the HFD control group compared with the NC group. Consumption of the HFD compared with the NC caused fatty liver abnormal mitochondria with loss of cristae, intramitochondrial granules, and a swollen and rarefied matrix. Administration of MMO significantly decreased body weight gain, and ALT, AST, and MDA levels; increased SOD activity and GSH-Px levels; alleviated fatty liver steatosis; decreased the early apoptosis population; downregulated SREBP-1c, Bax, Caspase-9, Caspase-3, TNF-α, and NF-κB protein levels; and upregulated PPAR-α, Bcl-2, and AMPK-α, compared with the HFD control group. MMO exhibited protective effects in mice with NAFLD by regulating the NF-κB anti-inflammation signaling pathways to inhibit inflammation, regulate AMPK-α, PPAR-α and SREBP-1c to improve lipid metabolism disorder, and regulate Bcl-2/Bax anti-apoptosis signaling pathways to prevent liver cell apoptosis. These results suggest that dietary supplementation with MMO ameliorates high-fat-diet-induced NAFLD.

## 1. Introduction

Non-alcoholic fatty liver disease (NAFLD) is the most common and prevalent liver disease, and hepatic lipid accumulation without excess alcohol consumption is the key feature [1]. NAFLD encompasses a series of liver disorders ranging from hepatic steatosis to nonalcoholic steatohepatitis, fibrosis, and cirrhosis, and eventually leads to hepatocarcinoma [2]. The worldwide prevalence of NAFLD is 11% to 46% [3], higher than that of type 2 diabetes and obesity [4,5]. Because of the high prevalence and subsequent risks, the diagnosis and treatment of NAFLD has become a major research focus.

Many factors, including lipid metabolic disturbance, insulin resistance, oxidative stress, and mitochondrial dysfunction, lead to the occurrence and aggregation of NAFLD [6]. Hepatic steatosis, however, is the first step toward NAFLD. The key feature of hepatic steatosis is the accumulation of triglycerides in the cytoplasm of hepatocytes, which makes the liver more sensitive and vulnerable to various insults [7]. Therefore, attenuating hepatic steatosis and improving hepatic lipid metabolism is a potential therapeutic strategy to prevent NAFLD progression. Sterol regulatory element-binding protein 1c (SREBP-1c), adenosine monophosphate-activated protein kinase (AMPK), and peroxisome proliferator-activated receptor α (PPAR-α), which contribute to maintaining the balance between hepatic lipid export and utilization, as well as lipid uptake and synthesis, play important roles in hepatic lipid metabolism though regulating their downstream gene expression. In addition, suppression of AMPK induces lipid accumulation in hepatocytes, and attenuates the ability of the mitochondria to oxidize free fatty acids, subsequently increasing the production of malondialdehyde (MDA) and reactive oxygen species (ROS), which cause inflammation and ultimately lead to the progression from NAFLD to NASH. Therefore, these functional factors are promising therapeutic targets for NAFLD [8,9].

Clinically, NAFLD patients with dyslipidemia are administered statins, which can improve liver function tests and steatosis. Further research is necessary to find drugs that ameliorate hepatic steatosis [10]. Marine organisms are important bioactive ingredients for pharmaceutical industries. Bioactive compounds can be isolated from marine plants, animals, and microorganisms [11].

Bioactive peptides isolated from jumbo squid inhibit lipid peroxidation in a linoleic acid model system [12]. The antioxidant tridecanoic peptide obtained from oysters (*Crassostrea gigas*) exhibits higher antioxidant activity than the synthetic antioxidant A-tocopherol against polyunsaturated fatty acid peroxidation [13]. Marine fish are rich in bioactive peptides and omega-3 polyunsaturated fatty acids that may act as anti-hypertensives and decrease systolic and diastolic blood pressure in patients with mild hypertension, high plasma cholesterol, low-density lipoprotein, very low-density lipoprotein, and triglycerides, while improving high-density lipoprotein levels [11]. Overall, several bioactive peptides and proteins obtained from marine organisms show great potential for use in nutraceuticals and pharmaceuticals. 

*Meretrix meretrix* widely distributes in the coastal areas of China, and is commonly served as a daily dish in China. It also exhibits medicinal activities, such as antioxidant [14], and anticancer [15]. In a previous study [16,17], *Meretrix meretrix* oligopeptides (MMO) with the amino acid sequence Gln-Leu-Asn-Trp-Asp were obtained from alcalase by hydrolysis processes. The underlying mechanisms of MMO against NAFLD were examined and explored. Treatment with MMO for 24 h increased the viability of NAFLD model cells by inhibiting apoptosis, alleviating oxidative stress, and improving mitochondrial dysfunction. Additionally, MMO restrained the activation of cell death-related pathways in NAFLD model cells compared with the control group. These findings indicate that MMO exerts protective effects against NAFLD in vitro. To the best of our knowledge, however, the effects of MMO on NAFLD have not been evaluated in vivo. Therefore, in the present study, we investigated the protective effects of MMO against NAFLD in mice.

## 2. Results

### 2.1. Effects of MMO on the Body Weight and Relative Liver Weight

**Figure 1:**
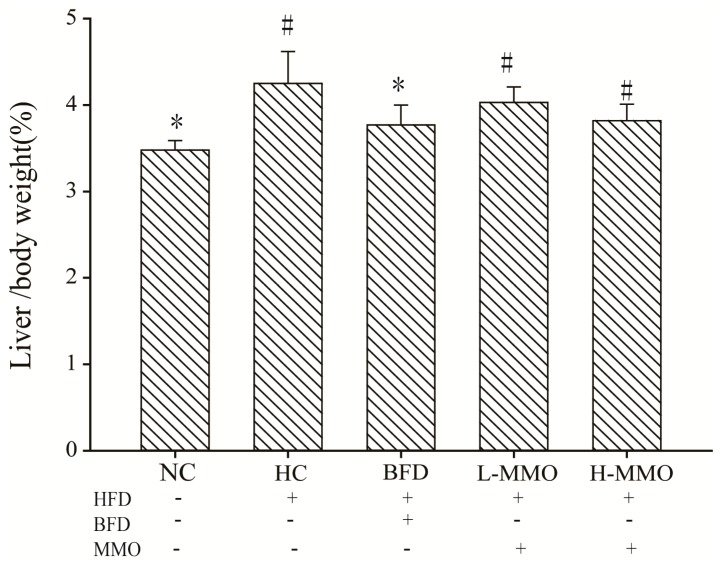
Effects of MMO on liver index (Liver weight/Body weight × 100) of NAFLD mice. Values are expressed as means ± SD. Values with different letters are significantly different in the groups (^#^ *p* < 0.05 compared with NC; * *p* < 0.05 compared with HC). Groups: NC = normal control; HC = HFD control; BFD = HFD + Bifendate; L-MMO = HFD + 50 MMO mg/kg; H-MMO = HFD + 250 MMO mg/kg.

**Figure 2:**
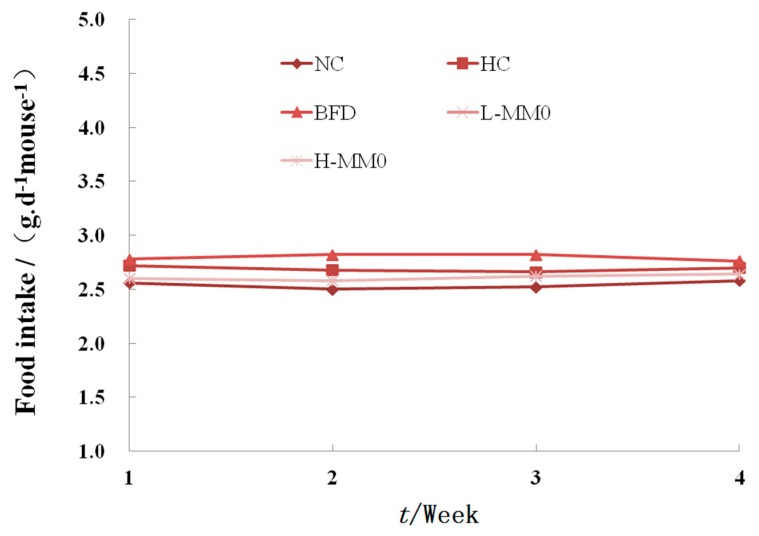
Effect of MMO on food intake of mice after four weeks treatment. LFD: low-fat diet; Values are expressed as means ± SD. Values with different letters are significantly different in the groups (^#^ *p* < 0.05 compared with NC; * *p* < 0.05 compared with HC). Groups: NC = normal control; HC = HFD control; BFD = HFD + Bifendate; L-MMO = HFD + 50 MMO mg/kg; H-MMO = HFD + 250 MMO mg/kg.

During six weeks of treatment, all animals survived. In mice fed a high-fat diet (HFD) as the HFD control group (HC) for six weeks, body weight increased from 9.55 g ± 2.02 to 15.27 g ± 3.11 and relative liver weight was higher than that in mice fed a normal diet. Mice fed an HFD and administered 50 or 250 mg/kg MMO, however, had a significantly lower body weight gain and relative liver weight than HC mice, as shown in Table 1 and Figure 1. Administration of bifendate (200 mg/kg) to mice fed an HFD diet also reduced body weight gain and relative liver weight compared with the HC group (*p* < 0.05). During the MMO treatment period, the food intake did not differ significantly among all the groups (Figure 2).

### 2.2. Effects of MMO on Liver Histopathology in Different Groups

**Figure 3:**
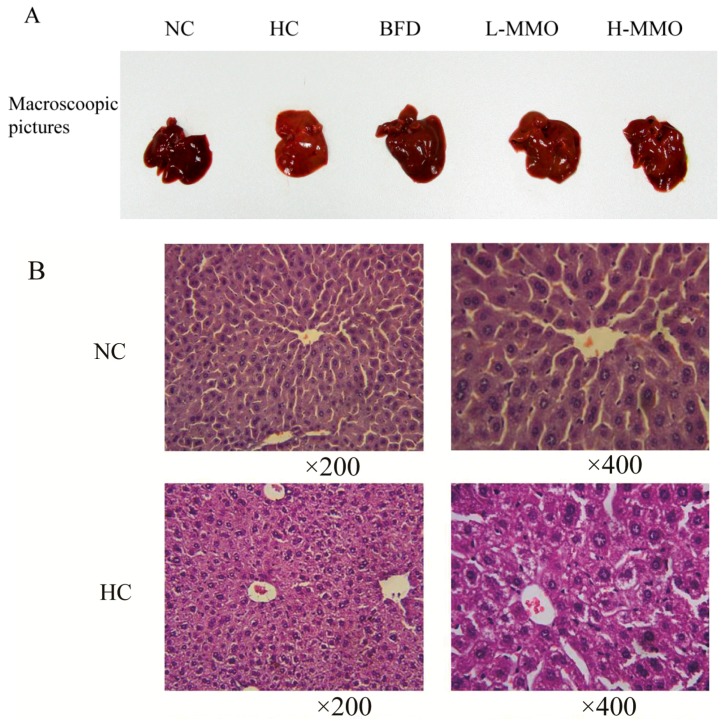

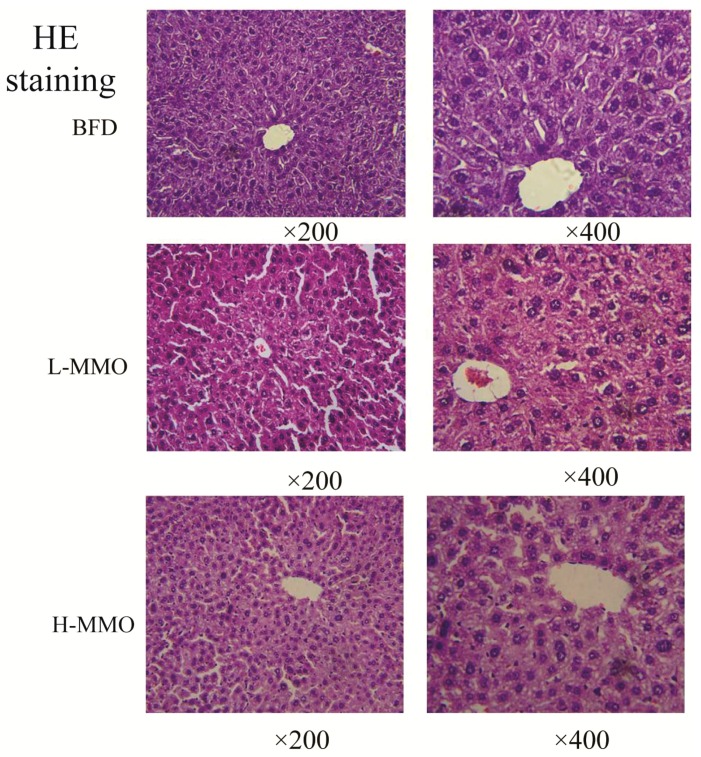
Macroscopic picture and HE staining liver (original magnification ×200 and ×400) from NC, HC, BFD, L-MMO and H-MMO. Groups: NC = normal control; HC = HFD control; BFD = HFD + Bifendate; L-MMO = HFD + 50 MMO mg/kg; H-MMO = HFD + 250 MMO mg/kg.

Liver sections from the NC group had a normal cell structure and lobular architecture (Figure 3). Liver specimens obtained from the HC group showed swelling and irregular hepatocyte arrangement in hepatocytes. Administration of MMO (50 and 250 mg/kg) or bifendate (200 mg/kg) modified the degree of swelling and irregular hepatocyte arrangement induced by the HFD.

### 2.3. Effects of MMO on the Ultrastructural Changes in the Liver in Different Groups

**Figure 4:**
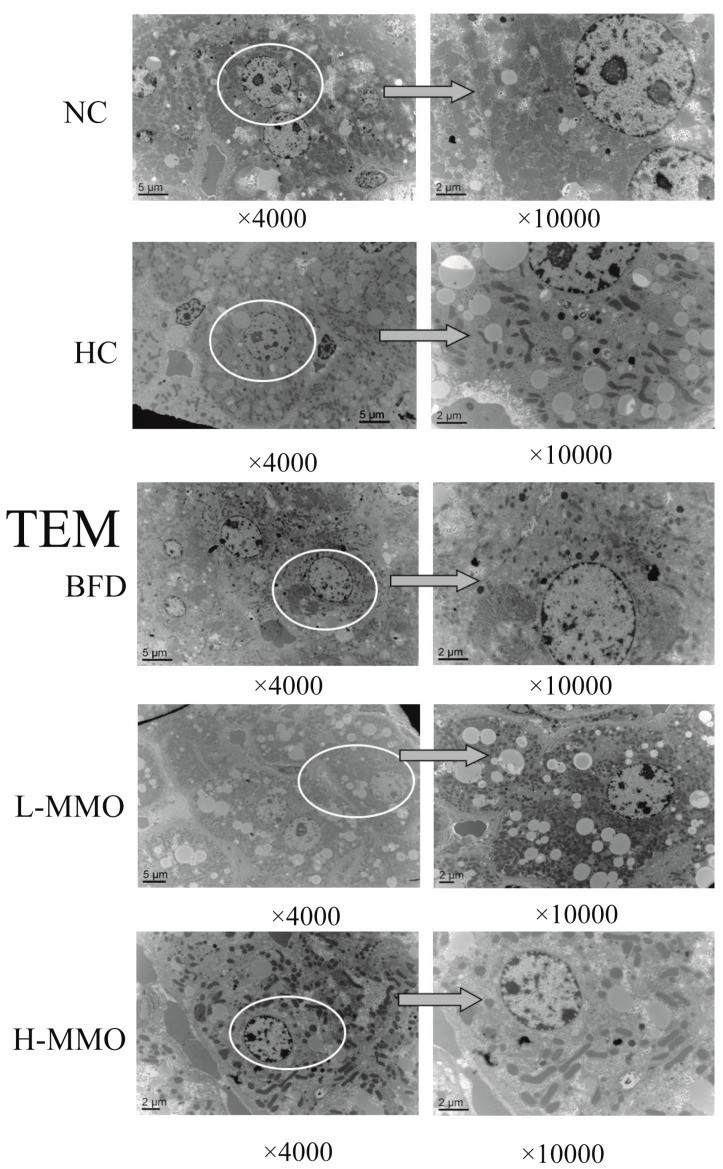
Electron microscopic study of hepatocytes (original magnification ×4000 and × 10,000) from NC, HC, BFD, L-MMO and H-MMO. The circles part and arrows means it was magnified from original magnification ×4000 to ×10,000. Groups: NC = normal control; HC = HFD control; BFD = HFD + Bifendate; L-MMO = HFD + 50 MMO mg/kg; H-MMO = HFD + 250 MMO mg/kg.

The development of hepatic steatosis is associated with altered mitochondrial morphology [18]. Ultrastructural changes of the mitochondria were further revealed by a transmission electron microscopy assay, and are shown in Figure 4. Ultrastructural observations revealed a reduced number of abnormal mitochondria with loss of cristae and intramitochondrial granules, swelling and rarefied matrix, and an increased number of lipid droplets in the HC group relative to the NC group. These mitochondrial alterations were clearly prevented by MMO and bifendate treatment. MMO and bifendate treatment clearly ameliorated HFD-induced changes to the liver ultrastructure based on the decrease of abnormal mitochondria and lipid droplets.

### 2.4. Effects of MMO on Serum Aspartate Transaminase (AST) and Alanine Transferase (ALT) Activities in Different Groups

**Figure 5:**
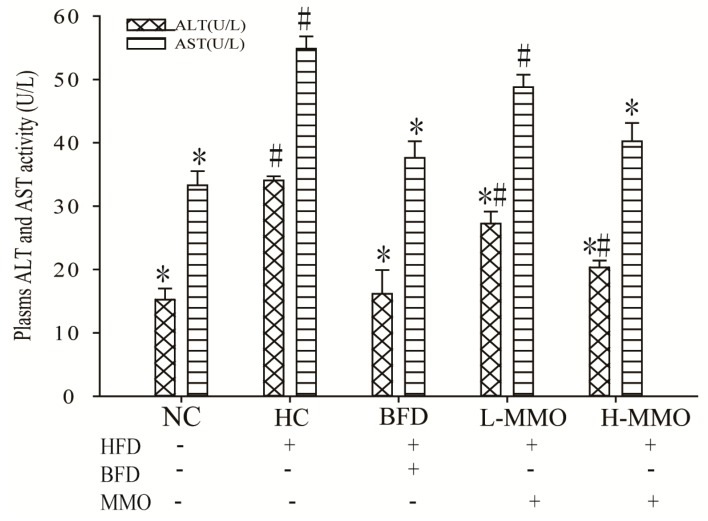
Effect of MMO on plasma level of AST and ALT of NAFLD mice. Values are expressed as means ± SD. Values with different letters are significantly different in the groups (^#^ *p* < 0.05 compared with NC; * *p* < 0.05 compared with HC). Groups: NC = normal control; HC = HFD control; BFD = HFD + Bifendate; L-MMO = HFD + 50 MMO mg/kg; H-MMO = HFD + 250 MMO mg/kg.

Increased AST and ALT activities in the serum are conventionally interpreted as a marker of liver damage. Serum AST and ALT activities are shown in Figure 5. AST and ALT activities in HC significantly increased consumption of an NC. Administration of 50 mg/kg and 50 mg/kg MMO or 250 mg/kg bifendate, however, significantly reduced AST and ALT activities consumption of an HC.

### 2.5. Effects of MMO on Antioxidant Capacities in Liver Tissues in Different Groups

**Figure 6:**
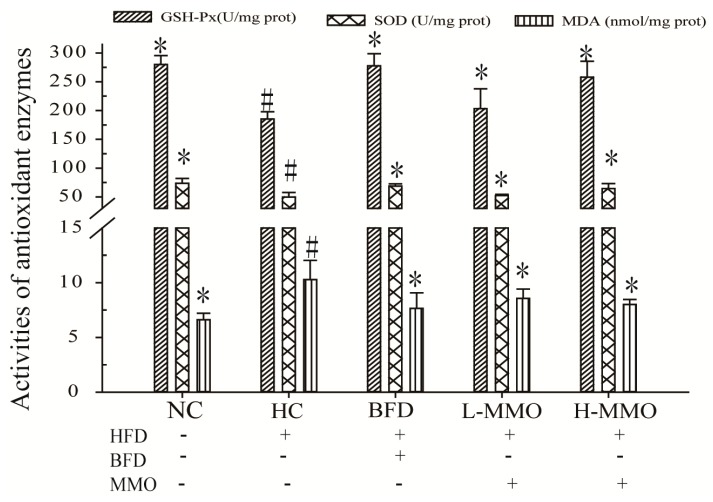
Effect of MMO on the activities of SOD, the levels of GSH-Px and MDA in liver tissues of NAFLD mice. Values are expressed as means ± SD. Values with different letters are significantly different in the groups (^#^ *p* < 0.05 compared with NC; * *p* < 0.05 compared with HC). Groups: NC = normal control; HC = HFD control; BFD = HFD + Bifendate; L-MMO = HFD + 50 MMO mg/kg; H-MMO = HFD + 250 MMO mg/kg.

Antioxidant capacities in the liver tissues are shown in Figure 6. Glutathione-peroxidase (GSH-Px) levels and superoxide dismutase (SOD) activities in the liver were significantly decreased, and MDA levels were significantly increased by 156.00% ± 2.96 in the HC group compared with the NC group. HFD mice treated with MMO (50 and 250 mg/kg) or bifendate (200 mg/kg) for four weeks had significantly higher GSH-Px levels and SOD activities, and decreased MDA levels compared with the HC group. The GSH-Px levels were not significantly different between HFD-fed mice administered L-MMO (50 mg/kg) and the HC group. 

### 2.6. Effect of MMO on Apoptosis in NAFLD Mice

**Figure 7:**
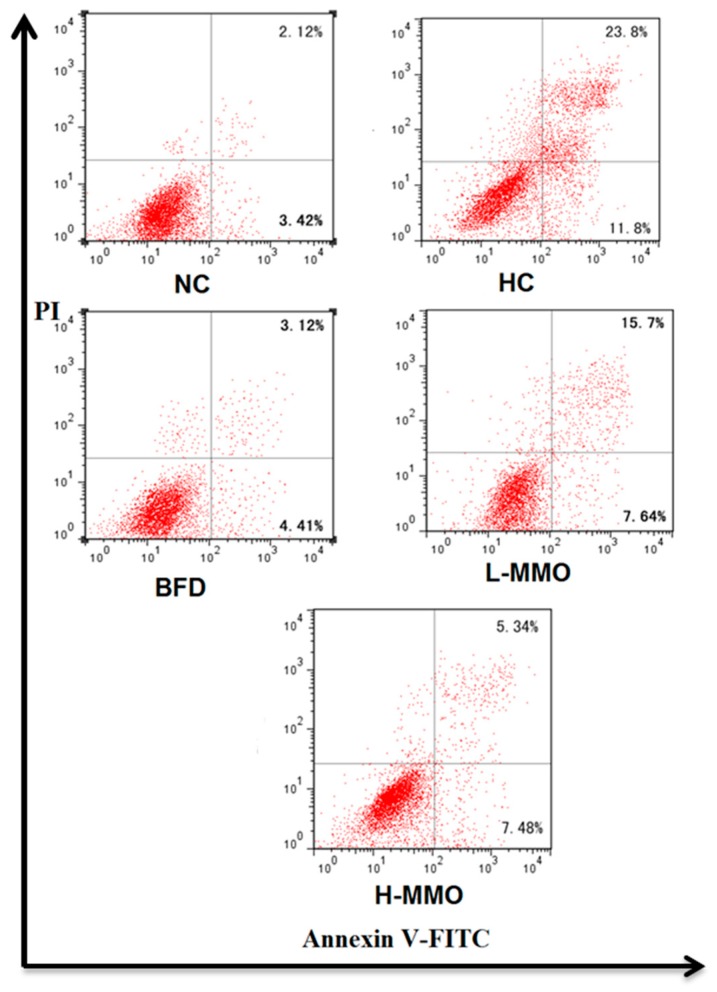
The apoptotic effects of MMO in liver tissues of NAFLD mice. UR represented late apoptotic ornecrosis cells (Annexin V^+^/PI^+^), LL represented normal cells (Annexin V^−^/P^−^), and LR represented early apoptotic cells (Annexin V^+^/PI^−^);Values are expressed as means ± SD. Values with different letters are significantly different in the groups (^#^ *p* < 0.05 compared with NC; * *p* < 0.05 compared with HC). Groups: NC = normal control; HC = HFD control; BFD = HFD + Bifendate; L-MMO = HFD + 50 MMO mg/kg; H-MMO = HFD + 250 MMO mg/kg.

Apoptosis of hepatocytes in the liver tissues are shown in Figure 7. It was evaluated based on Annexin V-FITC/PI staining. The early apoptosis population was significantly decreased when treated with MMO (50 and 250 mg/kg) or bifendate (200 mg/kg) for four weeks compared with the HC group. 

### 2.7. Effects of MMO on Hepatic Protein Expression Levels

**Figure 8:**
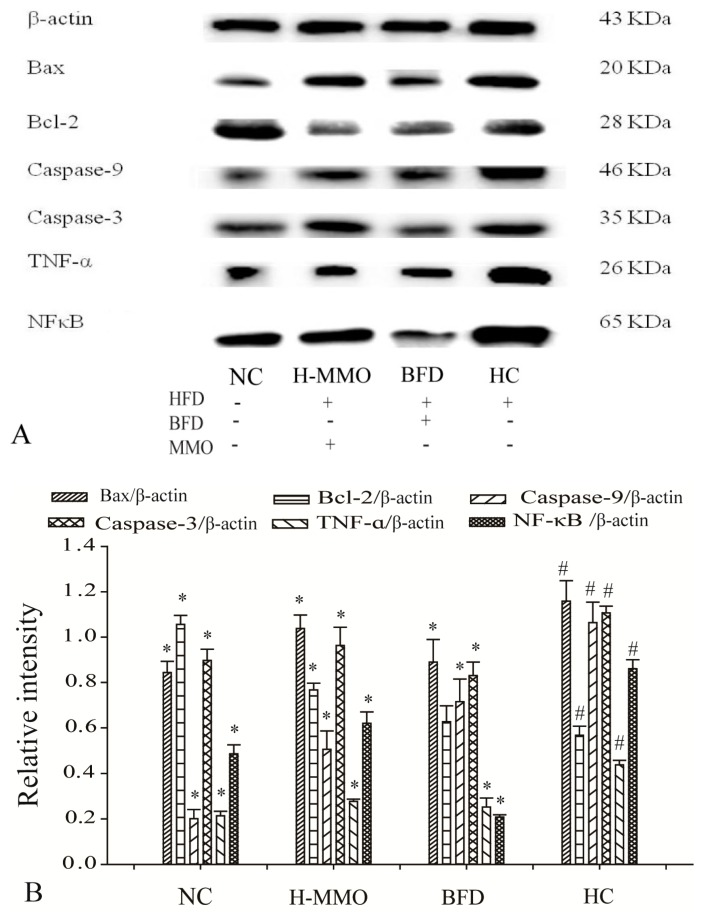

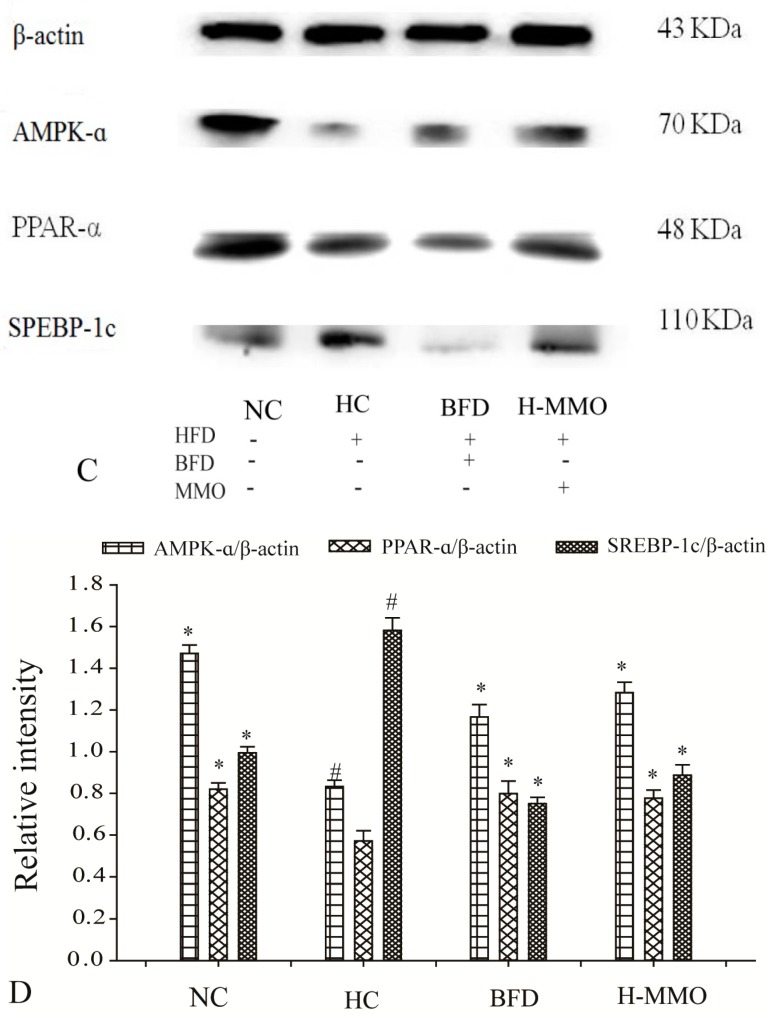
Effect of MMO on hepatic protein of key regulation involved in lipid metabolism, inflammation and apoptosis in liver tissues of NAFLD mice (**A**,**C**). Semi-quantitative analysis of Bax, Caspase-9, Caspase-3, TNF-α, Bcl-2 and NF-κB (**B**) was performed by calculating the density of Western blot bands. AMPK-α, SREBP-1c and PPAR-α (**D**) was performed by calculating the density of Western blot bands. Protein expression was analyzed by Western blot and normalized to β-actin. Values are expressed as means ± SD. Values with different letters are significantly different in the groups (^#^ *p* < 0.05 compared with NC; * *p* < 0.05 compared with HC). Groups: NC = normal control; HC = HFD control; BFD = HFD + Bifendate; H-MMO = HFD + 250 MMO mg/kg.

Bax, Caspase-9, Caspase-3, Tumor necrosis factor-α (TNF-α), and Nuclear factor-κB (NF-κB), and SREBP-1c expression levels increased, and Bcl-2, AMPK-α, and PPAR-α decreased in the HC group compared with the NC group (Figure 8). Additionally, Bax, caspase-9, caspase-3, TNF-α, NF-κB, and SREBP-1c expression levels decreased, and Bcl-2, AMPK-α, and PPAR-α increased in HFD mice administered MMO (50 and 250 mg/kg) or bifendate (200 mg/kg) compared with the HC group.

## 3. Discussion

In this study, we examined whether MMO improves hepatic lipid metabolism, antioxidative capacity, and protein expression of several genes in HFD-induced NAFLD mice. In a previous study [16], MMO exhibited antioxidant and anti-apoptotic effects in NAFLD model cells and therefore we hypothesized that MMO would exert protective effects against NAFLD in vivo in mice. 

Abnormal lipid metabolism from high-fat diets is the main cause of NAFLD. Excessive fat in diet directly leads to increased hepatic lipid synthesis with inflammatory responses [19,20,21]. Serum AST and AST levels are a sensitive marker of liver damage and increases of both in the blood indicate probable liver damage [22].

In the present study, we explored whether MMO ameliorates steatosis in an HFD-induced mouse model of NAFLD. Body weight gain in MMO groups was significantly decreased compared with the HC group. Administration of MMO decreased the abnormal mitochondria, and fat deposition in the liver tissue compared with the HC group. The ALT and AST activities were increased in the HC group, suggesting serious hepatocellular injury, consistent with the histologic results. Supplementation of the diet with MMO significantly attenuated the increased activities of the serum enzymes and liver damage. These findings indicate that MMO inhibits lipid accumulation and hepatic damage in the liver tissue.

An HFD increases oxidative stress in mammals [23]. Oxidative stress is crucially involved in the pathogenesis of NAFLD because the increased production of reactive oxygen species (ROS) leads to lipid peroxidation [24,25]. Lipid peroxide and hepatic MDA formation are commonly used as biomarkers of oxidative injury and liver tissue damage, respectively [26]. SOD and GSH-Px are important antioxidant enzymes present in the liver that prevent damage due to ROS generated in vivo during oxidative stress [27,28]. MDA is the product of lipid peroxidation and can eventually alter the biofunction of cells and cell membranes [29]. In addition, lipid accumulation in the liver frequently accelerates the release of MDA, which could promote nuclear-mitochondrial DNA damage and apoptosis by producing reactive ROS30. In the present study, the GSH-Px level and SOD activity decreased, and the MDA level increased in the HC group. MMO increased the GSH-Px level and SOD activity, and reduced the MDA level. Therefore, MMO administration improved the oxidative balance in HFD-induced NAFLD mice. 

Transcriptional factors such as AMPK-α, PPAR-α, and SREBP-1c were measured in the current study. AMPK-α and PPAR-α expression decreased and SREBP-1c expression increased in the HC group. In contrast, AMPK-α and PPAR-α expression increased and SREBP-1c expression decreased after MMO treatment. Those findings indicate that MMO could improve lipid metabolism disorder.

TNF-α is an important and pluripotent cytokine in host defense and inflammation by the activation of NF-κB and/or the apoptosis pathways [30]. The NF-κB pathway is one of the best-characterized signaling pathways in the pathogenesis of a wide variety of diseases, including liver diseases like NAFLD, inflammatory disorders, and tumor development [31,32]. Increased TNF-α expression leads to hepatic peroxidation and oxidative stress–induced damage. Subsequently, TNF-α stimulation activates the NF-κB signaling pathway, which is also an important transcription factor in the progression of NAFLD by mediating the cellular stress response [20]. 

Moreover, liver cell apoptosis promotes liver fibrosis. The Bcl-2 family, Bcl-2 and Bax, plays pivotal roles in regulating mitochondrial outer membrane permeability [33] The relative Bcl-2/Bax ratio may serve as a key sensory switch to dictate cell apoptosis [33,34]. Indeed, inhibiting liver cell apoptosis is related to a reduction in caspase-3 and caspase-9 and an increased Bcl-2/Bax ratio. Overexpression of Bcl-2 and decreased expression of Bax can enhance cell survival by suppressing apoptosis in a wide range of cells [35]. Therefore, inhibiting the activity of NF-κB in NAFLD as well as cell apoptosis is considered to be a therapeutic strategy against this disease. In the present study, expression of NF-κB and TNF-α was significantly decreased in animals administered MMO. Decreases in caspase-9 and caspase-3, and an increase in the Bcl-2/Bax ratio were observed in mice administered MMO. These results demonstrated that the protective effects of MMO on NAFLD are associated with its ability to regulate the NF-κB and Bcl-2/Bax signaling pathways to inhibit inflammation and liver cell apoptosis. The Annexin V-FITC/PI double staining assay supported our findings of suppressed apoptosis.

In conclusion, the findings of the present study indicated that MMO had therapeutic effects on HFD-induced NAFLD in mice. The protective effects of MMO in NAFLD mice are associated with its ability to regulate the NF-κB anti-inflammation signaling pathways to inhibit inflammation; regulate AMPK-α, PPAR-α, and SREBP-1c to improve lipid metabolism disorder; and regulate Bcl-2/Bax anti-apoptosis signaling pathways to prevent liver cell apoptosis. These results provide evidence that MMO is a promising drug candidate for the treatment of NAFLD. Further studies to elucidate the detailed mechanisms of the therapeutic effects of MMO will facilitate the discovery of treatment options for NAFLD.

## 4. Materials and Methods

### 4.1. Chemicals and Reagents

Antibodies against β-actin, Bcl-2 Associated X Protein (Bax), B-cell lymphoma-2 (Bcl-2), cysteinyl aspartate specific proteinase Caspase-3 (Caspase-3), cysteinyl aspartate specific proteinase Caspase-9 (Caspase-9), Tumor necrosis factor-α (TNF-α), Nuclear factor kappa B (NF-κB), AMPK-α, PPAR-α, SREBP-1c were purchased from ABGENT (San Diego, CA, USA) and other chemicals were obtained from Sigma (Shanghai, China). BCA Protein Assay Kits was obtained from Beyotime Institute of Biotechnology (Nanjing, China). Alanine aminotransferase (ALT) and Aspartate transaminase (AST) test kits were obtained from Nanjing Jiancheng Bioengineering Institute (Nanjing, China). GSH-Px, malonic dialdehyde (MDA) and Superoxide Dismutase (SOD) detection kits were purchased from Nanjing Jiancheng Bioengineering Institute (Nanjing, China). Annexin V-FITC/PI double Staining Kit (YTHX Biotechnology Co., Ltd., Beijing, China).

### 4.2. Preparation of Meretrix meretrix Oligopeptides (MMO)

The *Meretrix meretrix* was hydrolyzed by alcalase enzyme with the best hydrolysis condition based on an index of triglyceride content: temperature of 40 °C, time 8 h, pH 9.5, dosage 1000 U/g, and solid-liquid ratio of 1:2. *Meretrix meretrix* oligopeptides (MMO)’s amino acid sequence was Gln-Leu-Asn-Trp-Asp [17].

### 4.3. Animals, Diet and Study Design

The treatment and care of these mice were according to the Provisions and General Recommendation of Chinese Experimental Animals Administration Legislation. The animal protocol was approved by the Zhejiang province science and technology hall (Zhejiang Ocean University, Zhoushan, Zhejiang, China) (Permission No. 2016-0013). Fifty male mice (18–22 g) were purchased from the experimental animal center of Zhejiang Province (Hangzhou, Zhejiang, China) [Certificate No. SCXK (ZHE 2014-0001)], and were housed in air-conditioned quarters at controlled temperature (24 ± 1 °C) with 55 ± 5% relative humidity and 12 h light/dark cycles with free access to water.

Mice were divided into two groups: one group of 10 mice (ten mice per cage) was continuously fed with a standard diet for six weeks as the normal control group (NC). The other group of 40 mice were fed with fed a high-fat diet (HFD) containing 2.5% cholesterol, 0.5% bile sodium, 10% lard, and 87%standard diet (*w*/*w*), which was prepared by Zhejiang Medical Academy; food and water were provided ad libitum. 

After two weeks of HFD feeding to induce NAFLD, 40 HFD-fed mice were divided into four groups at random: HFD control group (HC), bifendate (positive control) group (200 mg/kg BW) (BFD), low dose of MMO group (50 mg/kg BW) (L-MMO), and high dose of MMO group (250 mg/kg BW) (H-MMO). The low dose of MMO and high dose of MMO were supplied with MMO by gavage. The four groups of mice were fed with HFD and weighed every three days and the food intake of mice in each cage was recorded daily for the next four weeks. After a four-week treatment with or without MMO and bifendate, blood samples were collected by removing the eyeball. The liver was carefully removed, weighed, and cut into several sections. One portion was fixed with 4% paraformaldehyde solution and the other portion was frozen in liquid nitrogen.

Blood samples were collected from the eyeballs in sterile tubes. Then the serum was separated by centrifuging the blood samples at 2200 *g* for 15 min.

The relative liver weight = liver weight (g)/body weight (100 g).

### 4.4. Biochemical Analysis of Serum Enzymes 

Biochemical analysis was carried out using AST (aspartate aminotransferase) and ALT (alanine aminotransferase) kits (Nanjing Jiancheng Bio-engineering Institute, Nanjing, Jiangsu, China). The activities of ALT and AST were determined according to the manufacturers’ instructions.

### 4.5. Histopathological Examination

Liver samples fixed in 4% paraformaldehyde solution were embedded in paraffin, sectioned to a 5 µm thickness, and stained with hematoxylin and eosin (H&E). The pathological changes were detected and photographed with an Olympus BX-51 microscope connected to a CCD-NC6051 camera shooting system.

### 4.6. Hepatocytes Apoptosis of Liver in NAFLD Mice

Livers were carefully removed from NAFLD mice, cut into several sections, washed with precooled saline, and dried with filter paper. Total of 0.2 g liver tissue was washed with phosphate-buffered saline (PBS) three times and ground gently with Stainless Steel Cell Cribble. Subsequently, the cells were washed twice with cold PBS and then resuspended in 1× binding buffer at a concentration of 5 × 10^5^ cells/mL. Ten microliters of PI and 5 μL Annexin V-FITC were added to each tube. Fifteen minutes later, 400 μL of 1× binding buffer was added to each tube at room temperature and in the dark. The samples were analyzed by flow cytometry (Millipore/Guava Technologies, Hayward, CA, USA).

### 4.7. Measurement of Glutathione Peroxidase (GSH-Px), Superoxide Dismutase (SOD) and Malondialdehyde (MDA) in Liver Tissue

The fresh liver from each mice was homogenized in physiological saline solution, the volume adjusted to 3 mL and centrifuged at 3000 rpm, 4 °C for 15 min. GSH-Px, SOD, and MDA in liver tissue were measured by using GSH-Px, SOD and MDA kits (Nanjing Jiancheng Bio-engineering Institute, Nanjing, Jiangsu, China), according to the instructions.

### 4.8. Transmission Electron Microscopy (TEM)

Livers were immediately cut into 5 mm × 5 mm pieces and placed into 2.5% glutaraldehyde and post-fixed in 1% phosphate-buffered osmium tetroxide. After being dehydrated, embedded, sectioned by Leica ultratome (Leica EM UC7, Wetzlar, Germany), and double-stained with uranyl acetate and lead citrate, observed captured with transmission electron microscope (FE-SEM, HITACHI, SU8010, Tokyo, Japan) (Hitachi Model SU-8010 SEM).

### 4.9. Western Blot Analysis 

Liver tissues were homogenized in RIPA buffer. Protein concentration wass measured by the BCA method (KeyGENbio, Nanjing, China). Protein samples were subjected to 12% SDS-polyacrylamide gels and then electrotransferred onto a PVDF membrane. After incubation with β-actin, Bax, Bcl-2, Caspase-3, Caspase-9, TNF-α, NF-κB, AMPK-α, PPAR-α, SREBP-1c primary, and secondary goat anti-mouse antibodies, proteins were visualized using ECL chemiluminescence (Beyotime, Shanghai, China). WB bands and quantitation were analyzed using Quantity One software (version 4.6.2) (Bio-Rad Laboratories, Inc., Hercules, CA, USA).

### 4.10. Statistical Analysis

Results were expressed as the mean ± standard deviation (S.D.) of at least three experiments. Statistically significant differences were determined using a one-way ANOVA with post hoc contrasts by Student–Newman–Keuls test by SPSS 19.0 software (SPSS, Chicago, IL, USA). Statistical significance was set at *p*-value < 0.05.

## Figures and Tables

**Table 1:** Effects of MMO on body weight of NAFLD mice.

Group	Dose/(mg/kg)	Initial Weight/g	Final Weight/g	Body Weight Gain/g
NC	-	22.86 ± 1.71	32.41 ± 2.68 *	9.55 ± 2.02 *
HC (HFD)	-	22.43 ± 1.76	37.69 ± 2.27 ^#^	15.27 ± 3.11 ^#^
BFD (HFD + Bifendate)	200	21.81 ± 2.20	31.83 ± 3.35 *	10.03 ± 2.29 *
L-MMO (HFD + MMO)	50	20.48 ± 2.31	32.89 ± 1.81 *	12.41 ± 2.31 ^#^
H-MMO (HFD MMO)	250	22.26 ± 1.83	33.10 ± 1.15 *	10.85 ± 1.83 *

^#^ *p* < 0.05 compared with NC; * *p* < 0.05 compared with HC.

## Abbreviations

The following abbreviations are used in this manuscript:

NAFLD	non-alcoholic fatty liver disease
MMO	*Meretrix meretrix* oligopeptides
ALT	alanine aminotransferase
AST	aspartate transaminase
MDA	malonic dialdehyde
SOD	superoxide dismutase
GSH-Px	glutathione peroxidase
SDS	sodium dodecyl sulfate
TNF-α	tumor necrosis factor-α
NF-κB	nuclear factor kappa B
SREBP-1c	sterol regulatory element-binding protein 1c
PPAR-α	peroxisome proliferator-activated receptor α
Bax	Bcl-2 associated X protein
Bcl-2	B-cell lymphoma-2
Caspase-3	cysteinyl aspartate specific proteinase -3
NC	normal control diet
HFD	high-fat diet
H&E	hematoxylin and eosin
TEM	transmission electron microscopy
